# Supplementary material for: HIVprotI: an integrated web based platform for prediction and design of HIV proteins inhibitors
Source: J Cheminform. 2018 Mar 9;10:12. doi: 10.1186/s13321-018-0266-y (PMC5845081; doi:10.1186/s13321-018-0266-y)
Supplement: Supplementary file 2 — Additional file 2. Source code of HIVProtI web server. [file 13321_2018_266_MOESM2_ESM.zip › HIVprotI_Source-code/hivprotI/weka-3-6-6/doc/weka/core/SystemInfo.html]

SystemInfo


---


|  |  |  |
| --- | --- | --- |
| **Overview** \| **Package** \| **Class** \| **Tree** \| **Deprecated** \| **Index** \| **Help** \| **Weka's home** | |  |
| **PREV CLASS**   **NEXT CLASS** | **FRAMES**    **NO FRAMES**     **All Classes** |  |
| SUMMARY: NESTED \| FIELD \| CONSTR \| METHOD | DETAIL: FIELD \| CONSTR \| METHOD |  |


---


## weka.core Class SystemInfo

```
java.lang.Object
  weka.core.SystemInfo
```

**All Implemented Interfaces:**: RevisionHandler

---

``` public class SystemInfo extends java.lang.Object implements RevisionHandler ```

This class prints some information about the system setup, like Java
version, JVM settings etc. Useful for Bug-Reports.

**Version:**
:   $Revision: 1.5 $

**Author:**
:   FracPete (fracpete at waikato dot ac dot nz)

---

**Constructor Summary**

`SystemInfo()`
  
          initializes the object and reads the system information


| **Method Summary** | |
| --- | --- |
| `java.lang.String` | `getRevision()`             Returns the revision string. |
| `java.util.Hashtable` | `getSystemInfo()`             returns a copy of the system info. |
| `static void` | `main(java.lang.String[] args)`             for printing the system info to stdout. |
| `java.lang.String` | `toString()`             returns a string representation of all the system properties |

**Methods inherited from class java.lang.Object**

`equals, getClass, hashCode, notify, notifyAll, wait, wait, wait`

**Constructor Detail**

### SystemInfo

```
public SystemInfo()
```

:   initializes the object and reads the system information


**Method Detail**

### getSystemInfo

```
public java.util.Hashtable getSystemInfo()
```

:   returns a copy of the system info. the key is the name of the property
    and the associated object is the value of the property (a string).

---


### toString

```
public java.lang.String toString()
```

:   returns a string representation of all the system properties

    :   **Overrides:**: `toString` in class `java.lang.Object`

---


### getRevision

```
public java.lang.String getRevision()
```

:   Returns the revision string.

    :   **Specified by:**: `getRevision` in interface `RevisionHandler`
    :   **Returns:**: the revision

---


### main

```
public static void main(java.lang.String[] args)
```

:   for printing the system info to stdout.


---


|  |  |  |
| --- | --- | --- |
| **Overview** \| **Package** \| **Class** \| **Tree** \| **Deprecated** \| **Index** \| **Help** \| **Weka's home** | |  |
| **PREV CLASS**   **NEXT CLASS** | **FRAMES**    **NO FRAMES**     **All Classes** |  |
| SUMMARY: NESTED \| FIELD \| CONSTR \| METHOD | DETAIL: FIELD \| CONSTR \| METHOD |  |


---
